# Bimetallic Mixed Clusters Highly Loaded on Porous 2D Graphdiyne for Hydrogen Energy Conversion

**DOI:** 10.1002/advs.202105235

**Published:** 2021-12-22

**Authors:** Yang Gao, Yurui Xue, Taifeng Liu, Yuxin Liu, Chao Zhang, Chengyu Xing, Feng He, Yuliang Li

*Adv. Sci*. **2021**, *8*, 2102777

https://doi.org/10.1002/advs.202102777

In the originally published article, the affiliations were wrong. Please find the correct affiliations here:

Y. Gao, Y. Xue, Y. Liu, C. Zhang, C. Xing, F. He, Y. Li

Institute of Chemistry

Chinese Academy of Sciences

Beijing, 100190, P. R. China

Email: xueyurui@iccas.ac.cn; hefeng2018@iccas.ac.cn; ylli@iccas.ac.cn

Y. Xue

Science Center for Material Creation and Energy Conversion

Institute of Frontier and Interdisciplinary Science

School of Chemistry and Chemical Engineering

Shandong University

Jinan 250100, P.R. China

T. Liu

The Education Ministry Key Lab of Resource Chemistry

Joint International Research Laboratory of Resource Chemistry

Ministry of Education, and Shanghai Key Laboratory of Rare Earth Functional Materials

College of Chemistry and Materials Science

Shanghai Normal University

Shanghai 200234, China

Email: liutf@shnu.edu.cn

Y. Gao, Y. Liu, C. Zhang, F. He, Y. Li

University of Chinese Academy of Sciences

Beijing 100049, P. R. China.

